# External Suction versus Water Seal after Selective Pulmonary Resection for Lung Neoplasm: A Systematic Review

**DOI:** 10.1371/journal.pone.0068087

**Published:** 2013-07-09

**Authors:** Tong Qiu, Yi Shen, Ming-zhao Wang, Yao-peng Wang, Dong Wang, Zi-zong Wang, Xiang-feng Jin, Yu-cheng Wei

**Affiliations:** Department of Thoracic Surgery, The Affiliated Hospital of Medical College, Qingdao University, Qingdao, China; Sapienza University of Rome, Italy

## Abstract

**Objective:**

To evaluate whether external suction is more advantageous than water seal in patients undergoing selective pulmonary resection (SPR) for lung neoplasm.

**Summary of Background Data:**

Whether external suction should be routinely applied in postoperative chest drainage is still unclear, particularly for lung neoplasm patients. To most surgeons, the decision is based on their clinical experience.

**Methods:**

Randomized control trials were selected. The participants were patients undergoing SPR with lung neoplasm. Lung volume reduction surgery and pneumothorax were excluded. Suction versus non-suction for the intervention. The primary outcome was the incidence of persistent air leak (PAL). The definition of PAL was air leak for more than 3–7 days. The secondary outcomes included air leak duration, time of drainage, postoperative hospital stay and the incidence of postoperative pneumothorax. Studies were identified from literature collections through screening. Bias was analyzed and meta-analysis was used.

**Results:**

From the 1824 potentially relevant trials, 6 randomized control trials involving 676 patients were included. There was no difference between external suction and water seal in decreasing the incidence of PAL [95% confidence interval (CI) 0.81−2.16; z = 1.10; P = 0.27]. Regarding secondary outcomes, there were no differences in time of drainage (95% CI−0.36−1.56, P = 0.22), postoperative hospital stay (95% CI -.31−.54, P = 0.87) or incidence of postoperative pneumothorax (95% CI 0.18−.02, P = 0.05) between external suction and water seal.

**Conclusions:**

For participants, no differences are identified in terms of PAL incidence, drainage time, length of postoperative hospital stay or incidence of postoperative pneumothorax between external suction and water seal. The bias analysis should be emphasized. To the limitations of the bias and methodological differences among the included studies, we have no recommendation on whether external suction should be routinely applied after lung neoplasm SPR. More high-quality randomized controlled trials are needed.

**Systematic Review Registration:**

None.

## Introduction

Chest drainage is the most important management method in pulmonary surgery. For lung neoplasm patients who undergo selective pulmonary resection (SPR), whether external suction should be applied is one of the major controversies. For most surgeons, the decision is made based on their experience. There are two contrasting viewpoints: (1) external suction appears to restore the negative intra-pleural pressure, eliminate residual space and expedite the fullest lung expansion as its major benefits [1]; and (2) non-suction, a water seal for example, is able to avoid the higher incidence of air leaks [2]. Generally, Lung neoplasm patients maintain better pulmonary function than severe emphysema or pneumothorax patients. We believe that the issue of suction should be analyzed independently for lung neoplasms rather than in association with other pulmonary air leak-associated diseases.

In the past years, many retrospective studies have trended toward routine non-suction management [3], [4]. Randomized control trials (RCTs) have reported different conclusions on this issue [5], [6]. Recently, investigators have focused on electronic devices with a controlled form of suction. This new system has been gradually popularized, but a general drainage system with or without suction should still be applied in the long term. The aim of this systematic review was to evaluate whether external suction was more advantageous than water seal in patients undergoing SPR for lung neoplasm.

## Methods

### Criteria for Considering Studies

We selected RCTs as the type of study. No language or publication date limits were set. The participants were patients undergoing SPR who were diagnosed with lung neoplasm. Studies including lung volume reduction surgery were excluded due to an initial association with poor pulmonary function. Pneumothorax studies were excluded because of the presence of air leaks. For the intervention, suction was compared with non-suction (water seal). Considering the different algorithms of postoperative management among institutions, we considered that suction beginning from chest closure during the operation [postoperative day (POD) 0] or from POD 2 was the same. The primary outcome was the incidence of persistent air leak (PAL). The definition of PAL was air leak for more than 3− days. The secondary outcomes included air leak duration, time of drainage, postoperative hospital stay and the incidence of postoperative pneumothorax.

### Search Methods for Identification

Two independent authors searched MEDLINE (http://www.ncbi.nlm.nih.gov), EMBASE (http://www.embase.com), and listed references. We also hand searched conference proceedings to identify published and unpublished trials. To minimize regional bias, we also searched the Chinese Biomedical Literature Database.

### Data Collection and Analysis

Titles and abstracts identified by the electronic and manual searches were evaluated by two independent reviewers. We carefully evaluated the identified studies to determine whether they met the inclusion criteria. Any disagreement was resolved by consensus. Complete information, including methods, participant characteristics, intervention, groups, results, and follow-up time, was entered in a paper form that was specifically designed for this purpose. Considering the potential heterogeneity of studies, we attempted only a narrative synthesis at this stage.

### Risk of Bias Assessment of the Included Studies

To estimate the validity of the included studies, the risk of bias in the results of each eligible study was assessed with domain-based evaluation according to the criteria of the Cochrane Handbook for Systematic Reviews of Interventions. The criteria for judging the risk of bias included random sequence generation and allocation concealment (selection bias), blinding of participants and personnel (performance bias), blinding of outcome assessment (detection bias), incomplete outcome data (attrition bias) and selective reporting (reporting bias).

### Statistical Analysis and Meta-Analysis

All the included studies were aligned to compare the characteristics of the participants, the interventions and the outcomes. The meta-analysis methods used for dichotomous and continuous data were Mantel-Haenszel and inverse variance, respectively. Heterogeneity was tested using the chi-square test (χ2 test). The meta-analysis was performed using a fixed-effects model when the heterogeneity test *p*>0.10. A random-effects model was used if heterogeneity existed, and the reasons for heterogeneity were analyzed. Descriptive analysis was implemented instead of meta-analysis if there was high heterogeneity or if there was no qualification to perform a meta-analysis. Odds ratios (ORs), relative risks (RRs) and mean differences (MDs) were the principal measures of effect and were presented as a point estimate with 95% confidence intervals (CIs) and P values in parentheses. Reviewer Manager 5.2.3 (The Cochrane Collaboration, Wintertree Software Inc., Canada) statistical software was used. We considered P values less than 0.05 to be statistically significant.

No protocol was used for this systematic review.

## Results

### Literature Screening and Risk of Bias

**Figure 1:**
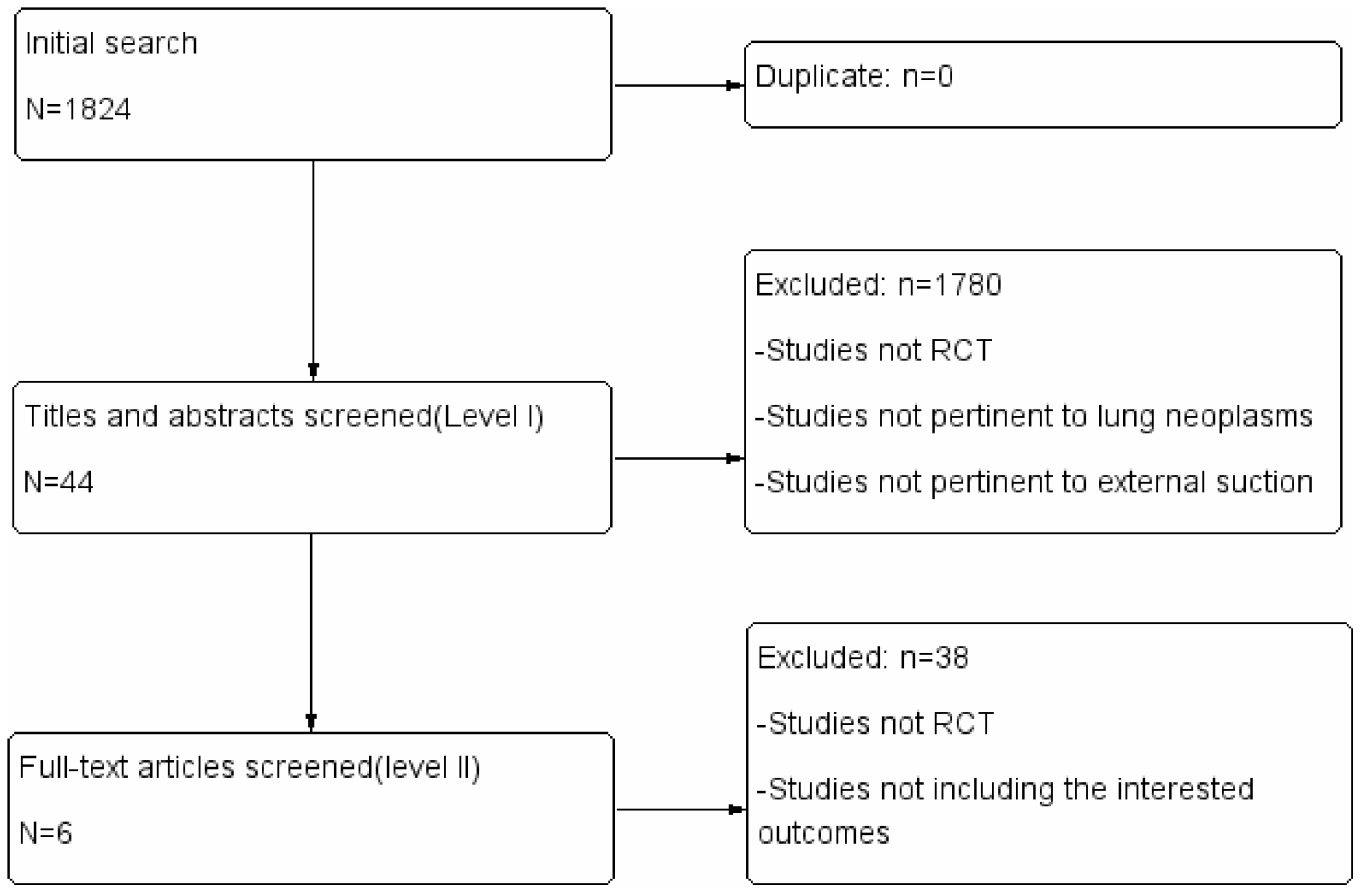
Flow chart of the literature screening.

A total of 1824 results were obtained with the search strategy from MEDLINE, EMBASE and the Chinese Biomedical Literature Database. After primary screening, 1780 articles were excluded. After secondary screening, 38 were excluded, and 6 RCTs were included [1], . A total of 676 patients were included in this analysis. The criteria and flow of selection and screening are shown in Fig. 1. The characteristics of the included studies are shown in Table 1.

**Table 1:** Characteristics of Included Studies.

Study	Participant	Suction pressure	Drainage system	Definition of PAL	Total cases	Pneumothorax cases	PAL cases
Cerfolio 2001	Air leak only	−20 cmH_2_O	Leak meter	>5 days	33	7	20
Marshall 2002	All	−20 cmH_2_O	Regular	None	68	4	None
Brunelli 2004	Air leak only	−20 cmH_2_O	Regular	>7 days	145	None	42
Alphonso 2005	All	−2 kPa	Regular	>5 days	239	5	21
Prokakis 2008	All	−(10–20) cmH_2_O	Regular	>3 days	91	4	12
Brunelli 2012	All	−(11–20) cmH_2_O	Digital	>7 days	100	None	9

PAL, persistence air leak

**Figure 2:**
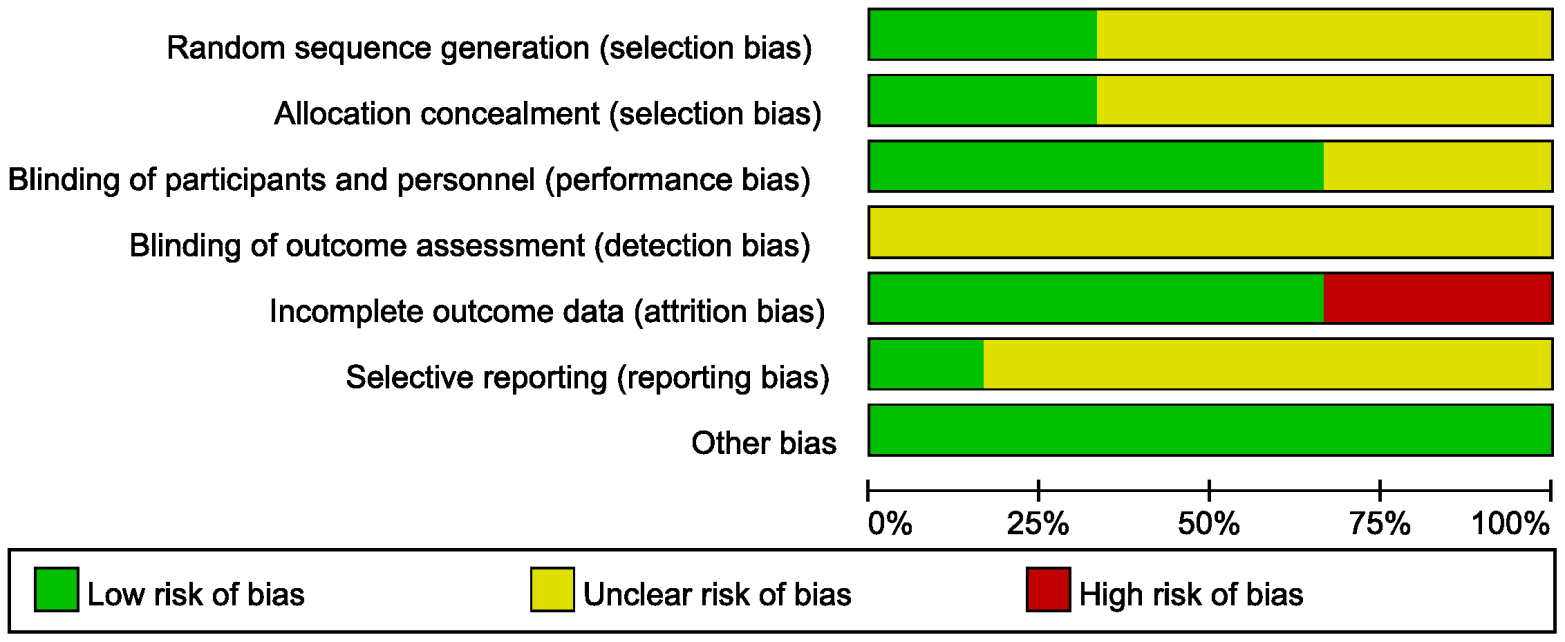
Graph of the risk of bias for the included studies. This graph is based on the results of domain-based evaluation according to the criteria of the Cochrane Handbook for Systematic Reviews of Interventions.

Two reviewers assessed the risk bias of the included studies. Alphonso (2005) and Cerfolio (2001) were found to have a high risk of bias due to incomplete data, while the other 4 studies were deemed unclear (Table 2). All the studies mentioned the blinding of surgeons; however, the blinding of outcome assessors in each study was not reported. Only one study was reported to be free of selective reporting, while freedom from selective reporting was not reported in the other 5 studies. The graph of risk bias for each included study is shown in Fig. 2.

**Table 2:** Risk of bias assessment.

Study	Sequence generation	Allocation concealment	Blinding of participants and personnel	Blinding of outcome assessment	Incomplete outcome data	Selective reporting	Other bias
Cerfolio 2001	Unclear risk	Unclear risk	Unclear risk	Unclear risk	High risk	Unclear risk	Low risk
Marshall 2002	Unclear risk	Unclear risk	Low risk	Unclear risk	Low risk	Unclear risk	Low risk
Brunelli 2004	Unclear risk	Unclear risk	Low risk	Unclear risk	Low risk	Unclear risk	Low risk
Alphonso 2005	Low risk	Low risk	Low risk	Unclear risk	High risk	Unclear risk	Low risk
Prokakis 2008	Unclear risk	Unclear risk	Low risk	Unclear risk	Low risk	Unclear risk	Low risk
Brunelli 2012	Low risk	Low risk	Unclear risk	Unclear risk	Low risk	Low risk	Low risk

### Meta-Analysis

**Figure 3:**
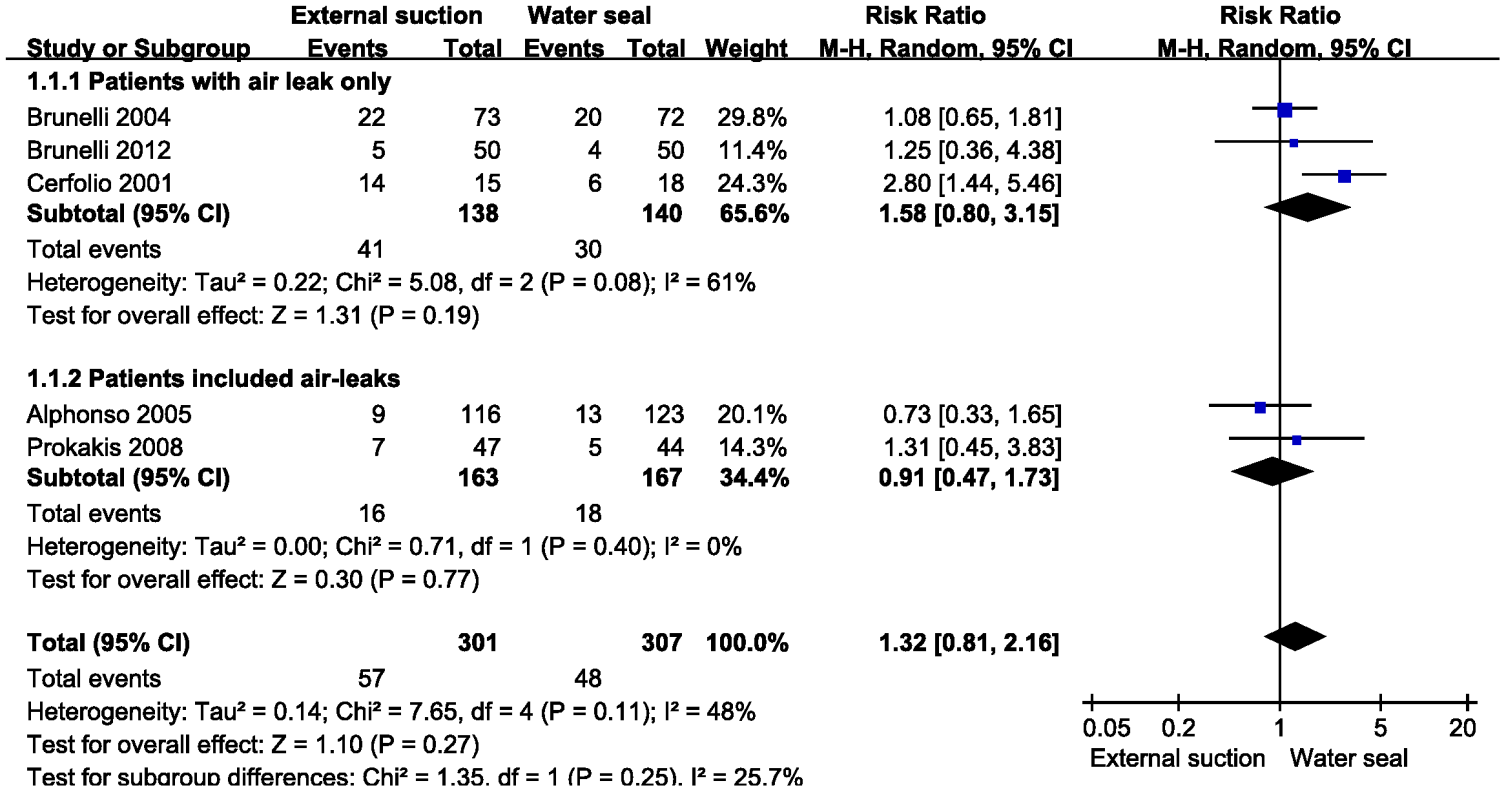
Meta-analysis of external suction versus water seal on the incidence of PAL. Subgroup 1: patients with air leak only. Subgroup 2: patients included air-leaks. M-H, Mantel-Haenszel. Events means patient with PAL.

Regarding the primary outcome, PAL incidence data were calculated from Brunelli (2004), Brunelli (2012), Cerfolio (2001), Alphonso (2005) and Prokakis (2008). According to the participant characteristics, the first 3 studies were placed into a subgroup of “patients with air leak only”, and the last 2 studies were placed into a subgroup of “patients included air leak” (Fig. 3). There was no difference between external suction and water seal in decreasing the incidence of PAL (total events: RR = 1.32, 95% CI 0.81−2.16, z = 1.10, P = 0.27). There was no statistical heterogeneity between the trials (χ^2^ = 7.65; df  = 4; P  = 0.11; I^2^ = 48%); therefore, a random-effects model was adopted.

**Figure 4:**
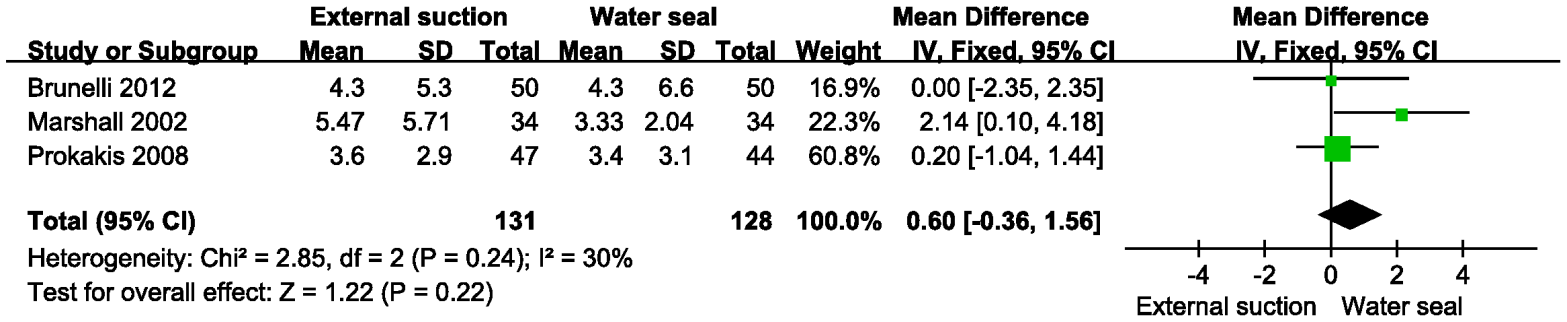
Meta-analysis of external suction versus water seal on the time of drainage. IV, inverse variance method.

**Figure 5:**
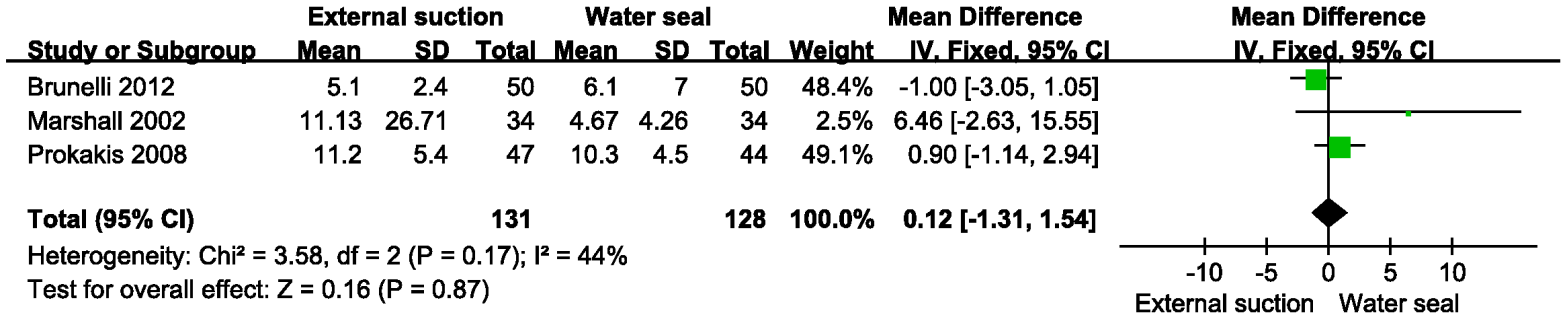
Meta-analysis of external suction versus water seal on the postoperative hospital stay. IV, inverse variance method.

**Figure 6:**
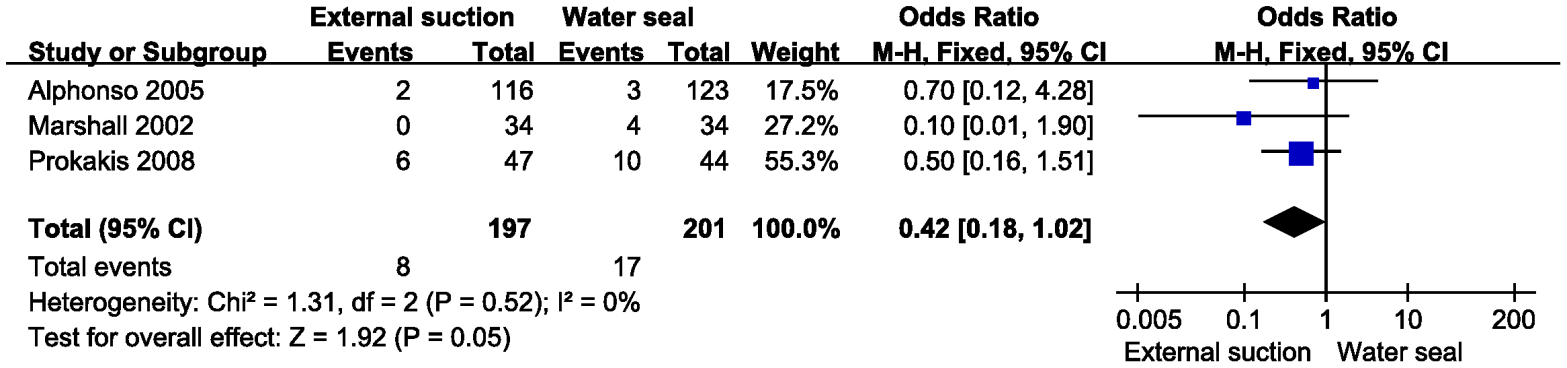
Meta-analysis of external suction versus water seal on the incidence of pneumothorax. M-H, Mantel-Haenszel method. Events means patient with postoperative pneumothorax.

The secondary outcomes time of drainage, postoperative hospital stay and postoperative pneumothorax were analyzed by meta-analysis. There was no statistical heterogeneity in these 3 parameters (fixed model, heterogeneity p>0.10). Because of a lack of data on the duration of air leaks, we excluded this item from the meta-analysis. Data on the drainage time was calculated from Brunelli (2012), Marshall (2002) and Prokakis (2008) (Fig. 4). The result showed no difference in decreasing the drainage time (MD = 0.60, 95% CI −0.36−1.56, z = 1.22, P = 0.22). Additionally, there was no difference in the postoperative hospital stay with the same studies (MD = 0.12, 95% CI −1.31−1.54, z = 0.16, P = 0.87) (Fig. 5). The postoperative pneumothorax data were calculated from Alphonso (2005), Marshall (2002), and Prokakis (2008) (Fig. 6) and showed no difference (OR = 0.42, 95% CI 0.18–.02, z = 1.92, P = 0.05).

## Discussion

Chest tube management is a basic skill for thoracic surgeons. One of the most interesting areas of focus is whether external suction should be applied to a chest drainage system. External suction applied after SPR was derived from lung volume reduction surgery based on clinical experience. Currently, many surgeons prefer external suction after lung neoplasm SPR as a routine procedure. In their opinion, external suction is able to maintain the negative pressure in the thoracic cavity, which reduces the residual cavity and promotes lung expansion. Therefore, external suction might reduce the incidence of persistent air leaks. Since 2000, several RCTs on this topic have been reported. Sanni *et al* reported a review of the published RCTs and indicated that no studies found in favor of suction to reduce the incidence of air leaks. However, new studies should be added [10]. Recently, investigators have focused on a new device with visually controlled suction pressure, which seems to be more beneficial for the natural physiology of the thoracic cavity. However, due to the portability and cost of this new product, a general drainage system with or without external suction is still the major issue in chest tube management, particularly in developing countries. Debate needs to be settled.

### Primary Outcome

PAL is still one of the most common postoperative complications. The common definition of PAL is a postoperative air leak sustained for more than 5 days [11]. Due to the publication time span of the included studies, we considered that the definition of PAL was air leak for more than 3–7 days. According to the result of the meta-analysis, there is no difference between external suction and water seal in decreasing the incidence of PAL. One of the reasons might be: Cerfolio RJ *et al* suggested that low pulmonary function is a key risk factor for PAL [12], while four of the included studies showed normal preoperative pulmonary functioning in patients [1], [6]–[7], [9]. According to the NCCN Guidelines for Non-Small Cell Lung Cancer, version 1.2013, a lung neoplasm with poor pulmonary reserve is appropriate for segmentectomy or wedge resection; however the patients in our study were underwent SPR. In our study, cases of pneumothorax, severe emphysema and other poor pulmonary function diseases were excluded, thus the quality of the lungs was relatively normal, and patients with lung neoplasm who underwent SPR were able to tolerate the reduction of pulmonary function. The other reason is that the application of staples or pulmonary air leak spot suturing is able to reduce the risk of PAL [13]. Furthermore, the application of surgical sealant during the operation decreases the potential risk of PAL [14], although none of the included studies stated using this technique.

### Secondary Outcomes

Regarding secondary outcomes, external suction provided no advantage over water seal in decreasing the time of drainage or postoperative hospital stay. In chest tube management, chest radiographs and observation of the chest drainage system are both necessary. In each included study, the indication for chest tube removal was similar. As shown in Fig. 4, a digital drainage system was used by Brunelli (2012), which may beneficially control pleural space pressure, while a general drainage system was used by Marshall (2002) and Prokakis (2008). Regarding the postoperative hospital stay, patients with air leak that persisted for more than 8 days were discharged with a Heimlich valve by Marshall (2002), while the other two studies performed no intervention at patient discharge.

External suction is a practical treatment for postoperative pneumothorax [15]. In our study, the results suggested that there was no difference between external suction and water seal in decreasing the incidence of postoperative pneumothorax. In recent years, two meta-analysis on suction versus water seal were published. Coughlin SM *et al* reported that water seal was associated with a significantly increased incidence of postoperative pneumothorax [16]. Deng B *et al* suggested that suction could reduce the occurrence of postoperative pneumothorax resulting from early air leak [17]. Through a careful review, we found that both studies included a study by Ayed AK in the term of postoperative pneumothorax. The weights of Ayed’s study in those two studies were 32.60% and 52.98%, respectively. Participants in Ayed’s research primarily underwent surgery for spontaneous pneumothorax, which was not in accordance with the inclusive criteria [18]. Thus, we excluded Ayed’s article during level 1 screening.

### Limitations

The PRISMA system was used to rate the overall quality of this research, but there are some limitations. One weakness is that there is no standard procedure in chest tube management. This vague management leads to different definitions of PAL and suction pressure. Additionally, which level of pneumothorax was confirmed as a complication was not mentioned in all included studies. Secondly, we should carefully consider the quality of the included studies. There was a high risk of bias due to incomplete outcome data in the studies of Alphonso (2005) and Cerfolio (2001). The heterogeneity test for each meta-analysis was well accepted. However, in Fig. 3, it should be noted that the subgroup heterogeneities were P = 0.08 and P = 0.40, respectively, while the total heterogeneity was P = 0.11. We found that Cerfolio (2004) may be the source of the heterogeneity (after excluding this study, the heterogeneity was P = 0.84). In our opinion, the small sample size of this study may impair the results. In this section, we used a random-effects model for meta-analysis and tolerated the heterogeneity. For the duration of air leak, only one included study reported the data. Therefore, more data sources are needed.

### Conclusion

For patients with lung neoplasm who underwent selective pulmonary resection, no differences were identified in terms of PAL incidence, drainage time, length of postoperative hospital stay or incidence of postoperative pneumothorax between external suction and water seal for chest tube management. The bias analysis of the included studies should be emphasized. To the limitations of the bias and methodological differences among the included studies, we have no strong recommendation on whether external suction should be routinely applied after selective pulmonary resection in lung neoplasm patients. A large-scale, well-designed RCT is needed to address this clinical issue.

## Supporting Information

Appendix S1 **Search strategy.**
(DOC)

Checklist S1 **PRISMA 2009 Checklist.**
(DOC)
