# Supplementary material for: External Suction versus Water Seal after Selective Pulmonary Resection for Lung Neoplasm: A Systematic Review
Source: PLoS One. 2013 Jul 9;8(7):e68087. doi: 10.1371/journal.pone.0068087 (PMC3706622; doi:10.1371/journal.pone.0068087)
Supplement: Appendix S1 — Search strategy. (DOC) [file pone.0068087.s001.doc]

**Appendix S1**

**Search strategy**

**MEDLINE**

#1 “Lung Neoplasms”[Mesh] OR carcinoma[Mesh], OR “Small Cell”[tiab] OR “Squamous Cell”[tiab] OR “Non-Small-Cell Lung”[tiab]

#2 lung[tiab] AND (cancer[tiab] OR neoplasm*[tiab] OR adenocarcinoma*[tiab] OR carcinoma*[tiab] OR tumor*[tiab] OR tumour*[tiab] OR squamous[tiab])

#3 (pulmon*[tiab]) AND (cancer[tiab] OR neoplasm*[tiab] OR adenocarcinoma*[tiab] OR carcinoma*[tiab] OR tumor*[tiab] OR tumour*[tiab] OR squamous[tiab])

#4 NSCLC[tiab] OR “nonsmall cell lung”[tiab] OR “non small cell lung”[tiab] OR sclc[tiab] OR “small cell lung”[tiab]

#5 (#1) OR (#2) OR (#3) OR (#4)

#6 resect*[tiab] OR lobetom*[tiab] OR excision[tiab]

#7 (#5) AND (#6)

#8 air leak*[tw]

#9 (#7) OR (#8)

#10 Suction[Mesh] OR Drainage[Mesh] OR “Chest tubes”[Mesh] OR “Pleural cavivity”[Mesh]

#11 suction*[tw] OR drainage*[tw] OR “pleural space*”[tw]

#12 (#10) OR (#11)

#13 (#9) AND (#12)

**EMBASE**

1 (lung tumor)/exp

2 exp Small Cell Carcinoma/

3 exp Squamous Cell Carcionma/

4 exp Lung non Small Cell Cancer/

5 (lung adj10 (cancer or neoplasm* or adenocarcionma* or carcinoma* or tumor* or squamous)).ti,ab.

6 (pulmon* adj10 (cancer or neoplasm* or adenocarcionma* or tumor* or tumour* or squamous)).ti,ab.

7 (NSCLC or nonsmall cell lung or non small cell lung or sclc or small cell).ti,ab.

8 1 or 2 or 3 or 4 or 5 or 6 or 7

9 (resect* or lobectom* or excision).ti,ab.

10 8 and 9

11 exp Lung Resection/

12 air leak*.ti,ab.

13 10 or 11 or 12

14 exp suction/

15 exp drainage/

16 exp chest tubes/

17 exp pleural cavity/

18 (suction* or drainage* or pleural space*).ti,ab.

19 14 or 15 or 16 or 17 or 18

20 13 and 19
